# Performance Testing of a Homemade Aerosol Generator for Pulmonary Administration of Dry Powder Formulations to Mice

**DOI:** 10.3390/pharmaceutics15071847

**Published:** 2023-06-28

**Authors:** Rick Heida, Paul Hagedoorn, Melle C. van Meel, Jurrie E. R. Prins, Frederike S. Simonis, Renate Akkerman, Anke L. W. Huckriede, Henderik W. Frijlink, Anne H. de Boer, Wouter L. J. Hinrichs

**Affiliations:** 1 Department of Pharmaceutical Technology and Biopharmacy, University of Groningen, 9713 AV Groningen, The Netherlands; r.heida@rug.nl (R.H.); p.hagedoorn@rug.nl (P.H.); r.akkerman@umcg.nl (R.A.); h.w.frijlink@rug.nl (H.W.F.); a.h.de.boer@rug.nl (A.H.d.B.); 2 Department of Medical Microbiology and Infection Prevention, University Medical Center Groningen, University of Groningen, 9713 AV Groningen, The Netherlands; a.l.w.huckriede@umcg.nl

**Keywords:** in vivo, pulmonary administration, dry powder formulations, spray drying, intratracheal administration, inhalation, rodents, aerosol, respiratory tract, in vivo imaging

## Abstract

A challenge in the development of dry powder formulations for inhalation is the poor reproducibility of their administration to small laboratory animals. The currently used devices for the pulmonary administration of dry powder formulations to small rodents often function sub-optimally as they use the same puff of air for both powder dispersion and aerosol delivery. As a result, either the air volume and flow rate are too low for complete powder deagglomeration or they are too high for effective aerosol delivery to the lungs of the animal. Therefore, novel and better devices are desired. We here present an aerosol generator designed to administer a pre-generated aerosol to the lungs of mice. By mapping the complex relationship between the airflow rate, delivery time and emitted dose, we were able to control the amount of powder being delivered from the aerosol generator. The emitted aerosol had a size range favorable for lung deposition and could be measured reproducibly. Nevertheless, in vivo fluorescent imaging still revealed considerable differences between the mice in terms of the dose deposited and the distribution of powder over the lungs, suggesting that a certain biological variation in lung deposition is inevitable.

## 1. Introduction

Dry powder drug or vaccine formulations for inhalation are of added value compared to liquid formulations as they show superior stability at ambient conditions (important for stockpiling) and can be administered conveniently and fast using (disposable) dry powder inhalers [1]. One of the major hurdles in the development of dry powder formulations for inhalation is that they cannot easily be evaluated in small laboratory animals. The reason is that most animals are obligate nose breathers. As a consequence, spontaneous inhalation generally results in poor lung deposition with a large fraction of the powder being deposited in the nasal–pharyngeal region. To overcome this, a range of devices have been developed for the intratracheal administration of powder formulations to anesthetized animals.

In this regard, the most widely used example has been the, now discontinued, Penn-Century Dry Powder Insufflator (Wyndmoor, PA, USA). However, with this system, powders are often inadequately dispersed as the dispersion and administration occur in one single maneuver using the same volume of air for both powder dispersion and pulmonary aerosol delivery [2,3,4,5,6,7]. Because of this, the volume that can be used for dispersion is limited by the tidal lung volume of the animal in order to not inflict damage to the animal during the administration process (i.e., potentially lethal damage that may also affect pulmonary absorption behavior). For mice, this is at maximum only 200 µL, which is far from sufficient for complete dose delivery and the proper dispersion of cohesive powders [7,8,9]. As a result, larger agglomerates occur in the delivered aerosol that cannot penetrate the small airways of the mice [2]. Also, to ensure delivery while using such a low amount of air and to enhance the powder dispersion process with the Penn-Century Insufflator, a high air velocity is required, which additionally contributes to poor deposition in the lungs because of inertial impaction to the walls of the upper respiratory tract [4,10,11]. Higher air volumes, up to 900 µL, have been used to increase the dispersion and amount of powder delivered [12], though this is even higher than the total lung capacity of mice (approximately 750 µL). Such high volumes are known to cause serious tissue damage in the lungs of mice. As a means to reduce these shortcomings, dispersion-enhancing excipients are often used for experiments with the Penn-Century Insufflator [13]. However, these excipients may not, or not to the same extent, be necessary nor wanted with the dry powder inhalers that are to be used for administration to humans. Another marketed device that shows a high resemblance to the Penn-Century Insufflator, and therefore likely has a comparable working principle, is the Powder Administration Device for Animals (Aptar Pharma, Crystal Lake, IL, USA). To our knowledge, however, no data have been published using this system. 

Over the years, various custom-made systems have been developed, often consisting of a small powder reservoir, a syringe and a needle only [12,14,15]. These systems, however, all have in common with the Penn-Century Insufflator the fact that they combine dispersion and administration in a single step. In addition, using such devices without a dispersion principle requires formulating the powder primarily for the delivery device to the animals instead of optimizing the formulation for the DPI that is to be used for administration to humans. A strongly improved alternative to the Penn-Century Insufflator for the active administration of dry powder aerosols to animals is the PreciseInhale^®^ (Inhalation Sciences, Huddinge, Sweden). This is a modular system that separates the aerosol-generation process (using either liquids or powders) from the administration into animals, which enables choosing the air volumes and velocities independently for both steps. Although generally good results have been achieved in powder characterization studies [13,16,17], for certain formulations the system was reported to be ineffective in reaching the desired dose due to agglomerate formation [16]. In addition, the pressures used for the dispersion of the powders into a suitable aerosol vary between 10 and 160 bar (1000 and 16,000 kPa) [18,19], which may result in particle damage, followed by a significant reduction in particle size, particularly for spray-dried powders.

We reported previously on the design of a home-made prototype aerosol generator for the administration of dry powder formulations to mice [10,11]. To address the limitations encountered using the Penn-Century Insufflator, we decided, similarly as for the PreciseInhale, to separate the dispersion process from the administration step and, thus, to improve the properties of the aerosol without harming the animal. This enabled us to use much higher volumes of air for the dispersion at an average maximum pressure of only 50 kPa, which is closer to the range expected for an inhaler used for human pulmonary drug delivery (generally from 2 to 6 kPa). By this means, we could eliminate the risk of changing the particle size distribution of the aerosol due to particle damage, as occurs in a high-pressure nozzle. In addition, we decided to incorporate the air classifier of a marketed dry powder inhaler (Novolizer^®^ multi-dose dry powder inhaler) in order to prevent the need for preparing formulations for animal studies that are different from those used in future clinical studies. Furthermore, our prototype system enabled control over the delivered dose by adjusting the administration time to the volumetric flow rate from the aerosol chamber and the computed aerosol concentration in that chamber. Although we were able to achieve whole-lung deposition in mice, we still considered the losses in the dose compartment too high. In addition, the deagglomeration efficiency of our prototype aerosol generator was rather low due to an unsuitable arrangement of the dispersion principle. Therefore, only a small fraction of the weighed powder became available as a usable aerosol [10,11]. 

In this study, we made several modifications to the design of the aerosol generator in order to improve its performance. Hereafter, we accurately measured the emitted dose from our improved aerosol generator in vitro. Several relevant parameters were studied and characterized, such as the particle size distribution of the aerosol, the aerosol chamber volume and the emitted dose across a range of applied airflow rates. This enabled us to investigate whether full command over the aerosol generation and emitted dose from the aerosol chamber could provide complete control over the drug deposition and distribution in the mouse lung too. Ultimately, the system was validated in mice using fluorescence imaging to assess the relative distribution of powder over the lungs. 

## 2. Materials and Methods

### 2.1. Powder Preparation by Spray Drying

A 2% (*w*/*v*) solution in MilliQ water of β-cyclodextrin (Royal Avebe U.A., Foxhol, The Netherlands) was prepared together with trileucine (Bachem AG, Bubendorf, Switzerland) as a dispersion aid [20,21,22] in a 24:1 weight ratio. For visualization purposes during the in vitro studies, the coloring agent Ponceau 4R (Warner-Jenkinson Europe, discontinued, Amersfoort, The Netherlands) was added to the solution to obtain a final concentration of about 0.01% (*w*/*w*). For the in vivo studies, 0.6% (*w*/*w*) of the fluorescent dye indocyanine green (ICG, TCI Europe N.V., Zwijndrecht, Belgium) was used instead. The solutions were spray-dried using a Büchi Mini Spray Dryer B-290 in conjunction with a B-296 dehumidifier (both from Büchi AG, Flawil, Switzerland). The drying procedure was performed at a feed rate of 3 mL/min using an NE-300 syringe pump (ProSense B.V., Oosterhout, The Netherlands) with a 60 mL syringe (Codan B.V., Deventer, The Netherlands). The inlet temperature was set to 120 °C at an atomizing gas flowrate of 601 Ln/h (leading to an outlet temperature of 58 °C under maximal aspiration). 

### 2.2. Powder Characterization

Scanning electron micrographs of the spray-dried powder were obtained using a JSM-6460 scanning electron microscope (JEOL Ltd., Akishima, Tokyo, Japan). Briefly, powder samples were fixated on an aluminum sample disk using double-sided carbon tape. Next, the samples were sputter-coated with a 10 nm layer of gold using a JFC-1300 auto fine coater purged with argon gas (JEOL Ltd. Japan). Images were acquired at 3000× and 5000× magnifications under high vacuum via a secondary electron detector using an acceleration voltage of 10 kV, a working distance of 10 mm and a spot size of 25. 

In order to assess the primary particle size distribution of the powder, laser diffraction analysis was performed with a HELOS BF diffraction apparatus using a RODOS dry powder disperser (both from Sympatec GmbH, Clausthal-Zellerfeld, Germany). For the dispersion, a pressure of 3 bar was used after it was confirmed through applying a range of pressures that this pressure was sufficiently high for the complete dispersion of our formulation into primary particles, whereas the particles themselves did not fragment into smaller fractions. The HELOS BF system was equipped with a 100 mm lens (R3), enabling the measurement of particles in the range of 0.1–175 µm. Preceding the measurements, a reference measurement was carried out. Next, a small amount of powder (±10 mg) was loaded on the rotating disk of the RODOS disperser. Measurements were initiated when the optical density passed 0.2% on channel 30 and were terminated after 3 s. The particle size distribution was calculated automatically based on Fraunhofer’s approximation theory for diffraction. Based on the resulting distribution curve, the X10, X50 and X90 values were derived. These values represent the cumulative volume undersize curve, in which X50 is the median diameter, i.e., the diameter for which 50% of the total volume is represented by particles smaller than the X50 and 50% of the volume by larger particles. For aerosols in which all particles have the same mass and shape factor, the volume and mass distribution curves are the same. 

### 2.3. Aerosol Generator Design and Working Principle

**Figure 1:**
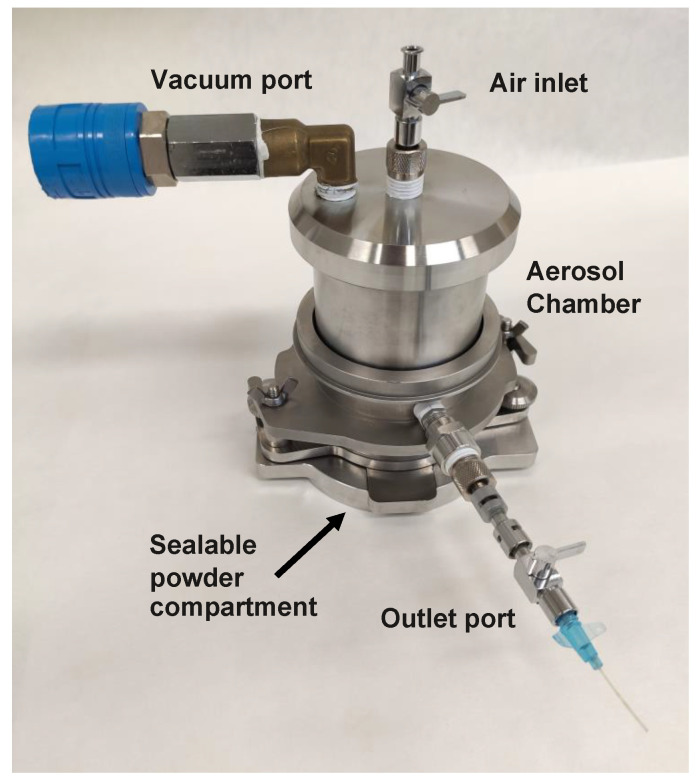
The aerosol generator. The system was composed of a sealable blister cup carrying spray-dried powder and the tortuous channel of the Turbuhaler^®^ dry powder inhaler for dispersion. Before operation, the pressure was lowered in the central aerosol chamber using a vacuum pump. After removal of the seal covering the blister, ambient air entrained the powder through the disperser into the aerosol chamber where the deagglomerated aerosol was stored. Hereafter, powder could be administered to the connected animal using a syringe pump at a preset flow rate.

The improved aerosol generator was a modular system (Figure 1), in which various parts, including the disperser and the dose compartment, could be exchanged. Also, the volume of the aerosol chamber could be changed using aluminum inserts. The types of dose compartment and disperser used here were comparable to those used for dry powder inhalers (DPI). This enabled the use of powders primarily formulated for use in a DPI and, additionally, to obtain comparable dispersion results with the aerosol generator. In this study, we used a sealable aluminum blister as the dose compartment (for approximately 50 mg powder) and the tortuous channel of the Turbuhaler^®^ DPI for the dispersion of the powder. Both were different from the prototype aerosol generator used in earlier studies that contained an open dose compartment and an air classifier as a disperser [10,11]. 

A detailed procedure of the assembly and operation of the aerosol generator is provided in Supplementary Method S1. In short, spray-dried powders were fed from a sealable powder compartment through a disperser that discharged the aerosol into a closed aerosol chamber. For the dispersion procedure, the pressure in the aerosol chamber was reduced using a standard rotary or piston vacuum pump. For this study, we used a Welch WOB-L^®^ 2522Z-02 piston vacuum pump (Welch vacuum, Fürstenfeldbruck, Germany) to create the desired pressure in the aerosol chamber (approximately 90 kPa below atmospheric pressure). After the pressure was lowered, a valve in the connector to the vacuum pump was closed. Upon the removal of the aluminum seal over the powder blister, ambient air was let in via the powder compartment through the disperser into the aerosol chamber. By this means, the air stream entrained the powder from the dose compartment and energized the disperser, similarly to the working principle of a DPI. For emitting the created aerosol from the aerosol chamber, an NE-300 syringe pump (ProSense B.V.) was used with a 60 mL syringe (Codan B.V.). An illustrative movie of the aerosolization process can be accessed via the Supplementary Information (Supplementary Video S1).

### 2.4. Assessing the Dispersion Efficiency of the Aerosol Generator

The particle size distribution of the emitted aerosol from the aerosol chamber was determined by laser diffraction analysis using a HELOS BF diffraction apparatus with an R3 lens (100 mm) with a measuring range from 0.1 to 175 µm. The HELOS was equipped with an Inhaler-2000 adapter (Sympatec GmbH, Clausthal-Zellerfeld, Germany) to conduct the aerosol effectively through the laser beam. For these measurements, aerosol was extracted from the aerosol generator by suction using a rotary vacuum pump attached to the (closed) Inhaler-2000 adapter instead of expelling the aerosol via the syringe pump. A calibrated venturi-meter was used to set the flow rate through the aerosol chamber into the Inhaler-2000 adapter to 30 L/min. Measurements were started at an optical signal of 1% on channel 30 and lasted for 10 s. Computations were based on the Fraunhofer optical model.

### 2.5. Determination of Emitted Dose

**Figure 2:**
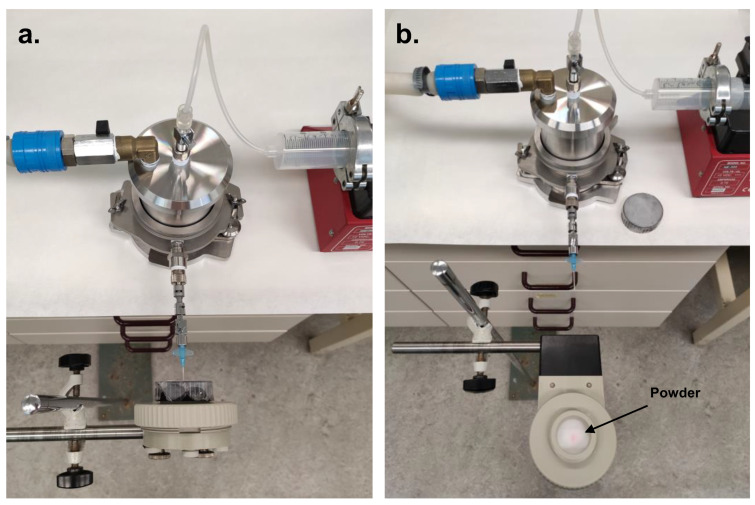
Filter set-up for determination of the emitted dose. (**a**) Top view showing the aerosol generator with the air inlet connected to the syringe pump for setting the flow rate and with the outlet port connected to the filter for powder collection; (**b**) after running the aerosol generator, the powder was collected from the filter for analysis of the emitted dose.

For the in vitro analysis of the emitted dose from the aerosol generator, a MicroGard^®^ II filter (Vyaire Medical GmbH, Hoechberg, Germany) was placed in front of the outlet port to collect the emitted aerosol. These filters were designed for the prevention of cross-contamination in the pulmonary function tests. They had a filtration efficiency for viruses and bacteria of 99.999% and yet at a very low resistance, causing a pressure drop of only 0.034 kPa at 30 L/min. Considering that most aerosol particles suitable for inhalation are within the same size range (1–5 μm) as bacteria (2–3 μm) and are even considerably larger than viruses (0.02–0.2 μm), we expected that the collection efficiency for aerosol particles would be the same. To verify this, we used a double-filter set-up in some experiments; by analyzing both filters separately, we could confirm that no particles passed the first filter, as we found no particles in the second. Two chamber volumes of 221 mL and 98 mL were tested by using either 0 or 2 insert cylinders in the chamber (Supplementary Figure S1f). To control the expelled airflow (with aerosol), the syringe pump was connected to the air inlet and set at a constant flow rate of 20 mL/min (Figure 2a). Immediately following the aerosol generation, the air inlet and powder outlet valves were opened, whereas the lower air channel towards the dose compartment and disperser was closed in order to not disturb the correspondence between the inlet and outlet valves. Next, the syringe pump was started to drive the aerosol through the outlet port into the filter during a 3 min runtime. A video of the aerosol being emitted from the cannula can be accessed via the Supplementary Information (Supplementary Video S2). 

After the runtime had elapsed, the syringe pump was switched off. Next, the filter was carefully removed from its holder (Figure 2b), and all the components (filter, filter holder and lid) were rinsed thoroughly with 5 mL of demineralized water to collect all the emitted powder. After sample collection, the emitted dose was analyzed by using the anthrone reaction, as previously described [23]. After the results showed that the small chamber volume, using 2 insert cylinders, yielded the highest emitted dose, the relationship between the flow rate and the emitted dose was determined for this condition using a fixed runtime of 3 min. Statistical analysis was performed in Graphpad Prism version 8.0.1. (Graphpad Software, San Diego, CA, USA) for the emitted dose as a function of the chamber volume using Welch’s *t*-test. A *p*-value ≤ 0.05 was considered significant. 

### 2.6. Validation of the Aerosol Generator for the Pulmonary Administration of Dry Powder Formulations to Mice

In vivo experiments were performed under the approval of the Central Committee for Animal Experimentation of the Netherlands (application number AVD1050020199104). For these experiments, in total, 12 female CB6F1 mice (8–10 weeks old) were used. Of these 12 animals, 6 were used to assess the powder deposition in and the distribution over the lungs. The remaining 6 were used for repeated administrations 2 and 4 days after the first administration experiment to assess whether the animals recovered well from a repeated administration procedure (using the same formulation). The animals that were followed-up were scored for general behavior and physical appearance and were weighed daily until sacrifice after the last administration. All animals were housed in groups of 3 with free access to food and water (standard diet). In order to minimize any autofluorescence, alfalfa-free bedding was used. 

**Figure 3:**
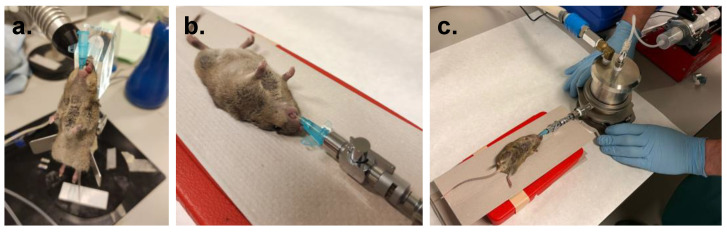
Aerosol generator operation in vivo. (**a**) Intubation procedure of an anesthetized mouse. Mice were intratracheally intubated using a 22 G cannula; (**b**) after generation of the aerosol, the intubated mice were connected to the system’s outlet port; (**c**) immediately after opening the air inlet valve and the output valve, the syringe pump was started, slowly administering the aerosol to the animals at a fixed flow rate during 3 min.

To ensure a sufficient fluorescence signal, we aimed for a dose of 3.75 mg/kg β-cyclodextrin, which equaled roughly 78.6 µg of powder (accounting for both trileucine and ICG). Using the equation from the flow-emitted dose relationship described in the results section, it was determined that administration of this dose required an expelling flow rate of 5.01 mL/min (83.5 µL/second) during a 3 min administration time. Mice were anesthetized via a subcutaneous injection with a solution of ketamine (75 mg/kg) and dexmedetomidine (1 mg/kg) and intubated intratracheally using a 22 G cannula (BD Insyte-W™ Peripheral Venous Catheter, BD, Franklin Lakes, NJ, USA) with a 0.9 mm outer diameter according to standardized procedures (Figure 3a) [24]. After intubation, the aerosol was generated according to the procedure described above. Immediately thereafter, the mice were placed in the supine position with the cannula connected to the aerosol generator (Figure 3b), and the syringe pump was run for 3 min to expel the aerosol through the cannula while keeping the air inlet closed (Figure 3c). 

After the syringe pump was stopped, the mice were gently disconnected from the system. Hereafter, the mice to be imaged were shaved around the chest region, after which they were placed inside a Lago X Imaging System (Spectral Instruments Imaging, Tucson, AZ, USA). To ensure the maximum signal from the chest region, the mouse paws were fixed using tape. Fluorescence emission intensity signals were subsequently measured at an excitation wavelength of 745 nm with an 810 nm emission filter specific to ICG (exposure time: 15 s; excitation power: 10; binning: 4) after a ‘region of interest’ (ROI) was defined for the measurement. Images were acquired and analyzed using the complementary Aura imaging software (Spectral Instruments Imaging). The signal intensities were expressed in terms of efficiency by automated comparison with the standard reference radiance, yielding a unitless fraction (between 0 and 1). Next, the total efficiency for the ROI was computed as the product of the mean efficiency of the defined ROI and the area of that ROI. After imaging the whole body, the mice were sacrificed, and the lungs were dissected for imaging the lung distribution in greater detail. In order to compare regional distribution, the lungs were further separated into individual lung lobes and analyzed again. The colon served as a control for background fluorescence. In between the administrations to individual mice, the aerosol chamber and outlet port (including the cannula) were rinsed with water and ethanol. Pressurized air was used to dry the components. Directly after administration, the animals that were followed-up for the repeated administration were injected with 1 mg/kg of atipamezole to antagonize the sedative effect of dexmedetomidine. Ophthalmic ointment was applied to prevent drying-out of the eyes.

## 3. Results

### 3.1. Characterization

#### 3.1.1. Particle Characterization

As shown by the SEM images, the spray drying resulted in particles with a slightly wrinkled surface (Supplementary Figure S2). The size distributions of the primary particles and of the particles emitted from the aerosol generator, as measured by laser diffraction, are presented in Table 1 for a 50 mg dose. The primary spray-dried particles had a median diameter (X50) of nearly 2 μm and a fraction < 5 µm approaching 100%. Laser diffraction diameters are different from the aerodynamic diameters that are generally obtained for inhalation powders with a cascade impactor analysis. For most crystalline particles, the aerodynamic diameters are generally slightly larger than the laser diffraction diameters. However, for spray-dried particles that approach sphericity and mostly have densities smaller than 1 g/cm^3^, the opposite is true and aerodynamic diameters are smaller, suggesting that the powder is suitable for inhalation. The aerosol emitted by the aerosol generator without inserts (221 mL) had a size distribution close to that of the primary particle size distribution, which appeared to not be affected by the insertion of concentric cylinders to reduce the aerosol chamber’s volume. However, after the removal of the outlet port (enabling measuring the particles directly from the aerosol chamber), we observed a rightward shift of the size distribution curve corresponding to the appearance of larger particles. Therefore, although complete dispersion of the powder in the aerosol generator was not obtained, the system had a classifying function that enabled the effective retention of particles larger than approximately 5 μm (for flow rates up to 30 L/min).

#### 3.1.2. Effect of Chamber Volume on the Emitted Dose

**Figure 4:**
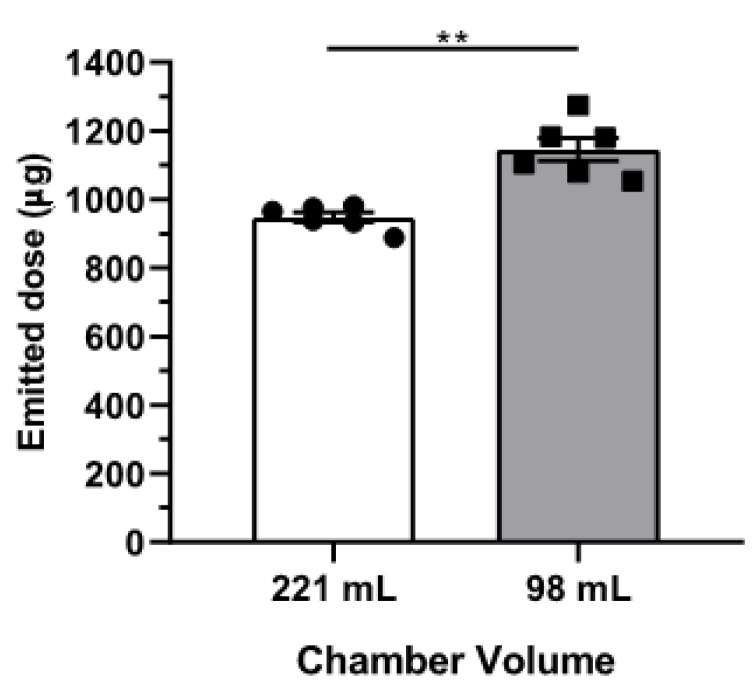
Emitted dose for two different chamber volumes, 221 mL (without inserts) and 98 mL (with inserts), after a runtime of 3 min at a flow rate of 20 mL/min. n = 6 per volume. Statistical analyses were performed using Welch’s *t* test (** *p* < 0.01).

The insertion of concentric cylinders in the aerosol chamber to reduce its volume was used to determine whether the resulting increase in the aerosol concentration led to a higher emitted dose over the same delivery time and at the same flow rate. Figure 4 shows the emitted dose at an expelling flow rate of 20 mL/min and a runtime of 3 min for the two chamber volumes used in this study (221 mL and 98 mL). The emitted dose increased significantly when the aerosol generator chamber volume was decreased (mean emitted dose of 947 ± 14 without inserts and 1145 ± 34 µg with inserts). However, the difference in the emitted dose for the different chamber volumes was smaller than was expected from the difference in the aerosol concentration. While the concentration as a result of reducing the aerosol chamber volume increased by a factor of 2.26, the emitted dose did not. There may be different reasons for this discrepancy, including an increased level of particle–wall contact in the chamber with the small volume. Also, an increased velocity of the air circulation in the chamber and the higher particle concentration resulting from the volume decrease may have affected the sedimentation behavior. The net effect of this complex interplay of variables may have also depended on the properties of the formulation and, therefore, there likely was an optimum in the chamber volume of the aerosol generator. Nevertheless, based on these delivery results, we decided to use the small chamber (98 mL) for all the following experiments.

#### 3.1.3. Emitted Dose as Function of the Expelling Airflow Rate

**Figure 5:**
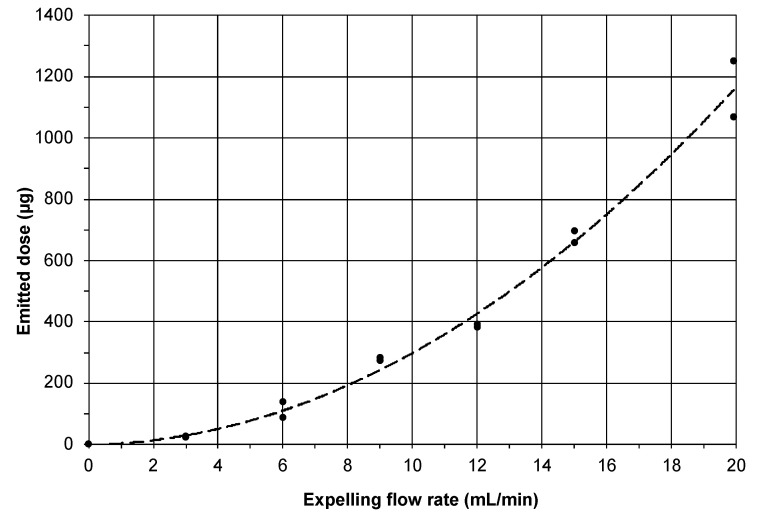
Emitted dose as a function of the expelling flow rate for a fixed runtime of 3 min. n = 2 for all investigated flow rates.

Figure 5 shows the emitted dose as function of the expelling flow rate, which was varied between 3 and 20 mL/min. Curve fitting along the duplicate measurements yielded the following quadratic equation: $y=28451x^{2}+1429x$ (R^2^ = 0.997), in which x equals the expelling flow rate and y equals the emitted dose. It can be seen that the emitted dose increased with an increasing expelling flow rate. The non-linear relationship, however, seemed to be the result of a complex interplay between the variables, including the effects of the aerosol dilution rate, residence time, air circulation velocity in the chamber and particle sedimentation. Although the exact interplay between the variables that determined the emitted dose remains largely unknown, the good agreement between the duplicate experiments at each flow rate made the relationship highly useful for predicting the emitted dose at a specific expelling flow rate. Therefore, the equation was used to compute the flow rate needed for the delivery of powder via the cannula to mice in the in vivo study.

### 3.2. In Vivo Validation

**Figure 6:**
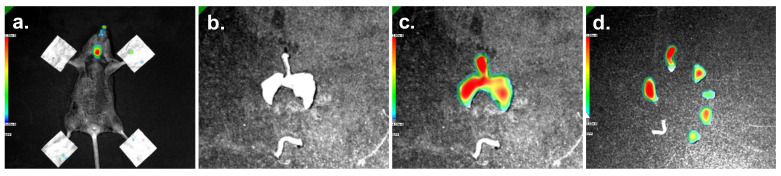
Fluorescence images showing the distribution of the ICG-labeled powder formulation from the aerosol generator over the lungs of a mouse. The powder was delivered at a fixed flow rate of 5.01 mL/min for 3 min, and mice were imaged immediately thereafter. (**a**) Mouse 5 shown (as a representative example) before dissection. The paws of the mice were fixed using tape to ensure that the body posture did not affect imaging; (**b**) dorsal view of the dissected lungs from the same mouse; (**c**) fluorescence overlay with the distribution of the compound over the trachea and the lung lobes; (**d**) dorsal view after separating the lungs into individual parts, starting from the top in clockwise direction: trachea, superior lobe, middle lobe, inferior lobe, post-caval lobe and left lobe. Fluorescence intensity values are shown in colors with red representing the highest intensity and blue the lowest. Scale bars represent fluorescence efficiency units.

To validate the applicability of the aerosol generator for in vivo delivery, powder was administered to the intubated mice at an expelling flow rate of 5.01 mL/min from the aerosol chamber for a 3 min runtime. Six mice were sacrificed directly after the first administration for imaging purposes. The fluorescence images of mouse 5 (as a representative example) showed the powder deposition results (Figure 6). When imaging intact mice (Figure 6a), fluorescence was mainly found in the trachea and was highly variable among the different animals. Therefore, we concluded that whole-mouse imaging may not be sufficiently reliable to determine the pulmonary powder deposition. For this reason, we removed the lungs from the mice (Figure 6b), showing that the powder was well-distributed over the entire lungs. After further dissection of the lungs into individual lung lobes (Figure 6c), it became convincingly apparent that the powder could penetrate deep into the lungs. A complete overview of all the mice is shown in Supplementary Figures S3–S5. 

**Figure 7:**
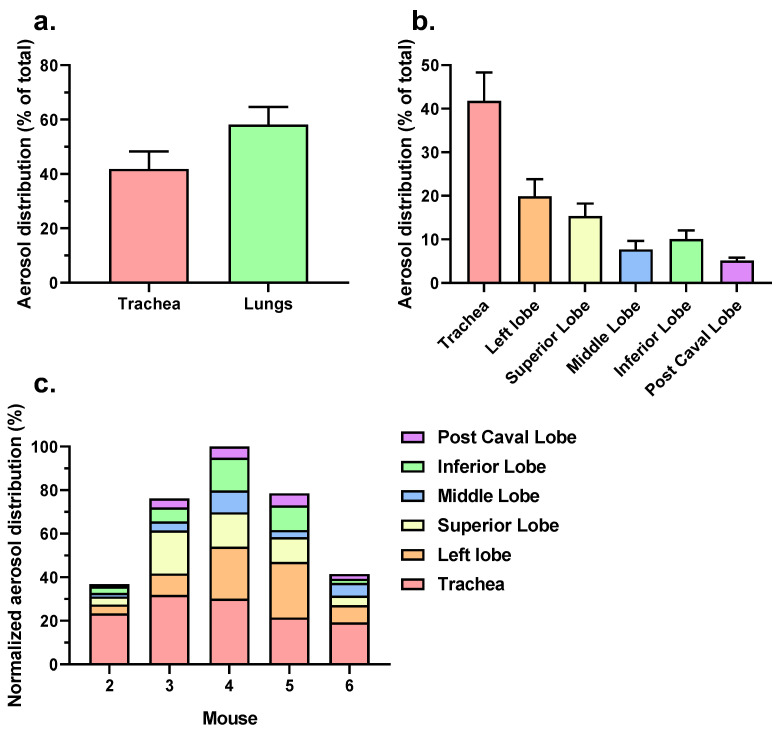
Distribution of the ICG-labeled powder deposition in the lungs derived from computed total efficiency values. (**a**) Comparison of trachea and lung deposition, both expressed as average percentage (±S.E.M.) of total deposition; (**b**) regional powder deposition over different lung lobes and the trachea as average percentage (±S.E.M.) of total deposition; (**c**) normalized regional powder deposition, taking mouse 4 with highest total deposition as reference (equated to 100%). Mouse 1 was excluded due to a dissection-related artifact. n = 5.

Although the fluorescence efficiency could not serve as a quantitative measure for the deposited powder mass, the emitted signal allowed estimating how the powder aerosol was distributed over the trachea and the different lung lobes and comparing the distribution among the different animals. The regional aerosol distribution is presented in Figure 7a,b. For the purpose of assessing the variation in the distribution between the individual mice, Figure 7c shows the normalized aerosol distribution relative to the animal with the highest total deposition (mouse 4 equated to 100%). Based on the fluorescence signals of four out of the five mice (except mouse 2), the powder deposition was higher in the lungs than in the trachea (Figure 7a). Powder was deposited in all of the lung lobes, with most of the powder being deposited in the more proximal sections of the lungs (Figure 7b). However, the total amount of powder distributed over the various lung lobes appeared to vary between the mice, with mouse 4 showing the highest aerosol deposition and mouse 2 the lowest (Figure 7c). This may have been caused by variations in how the tip of the cannula was positioned relative to the carina of the intubated animal and by biological variations related to breathing. Mouse 1 was excluded from the analysis due to a dissection-related artifact. 

The remaining six animals were administered again 2 and 4 days after the first administration; all animals survived the procedure. In addition, these mice did not show any deviations in physical appearance and behavior except for some weight loss (average 5.7 ± 3.3% relative to their starting weight), which can likely be attributed to the repeated exposure of the animals to the anesthesia regimen. 

## 4. Discussion

To eliminate, or at least minimize, some of the drawbacks that are inherent in using the currently available devices for the pulmonary administration of dry powder formulations to laboratory animals, we developed and tested an improved version of our previously described aerosol generator [10,11]. Various improvements were made compared to the first prototype of our aerosol generator and the Penn-Insufflator device. In summary, and most importantly, we replaced the dispersion principle for the aerosol generation and moved it from the top of the aerosol chamber to the bottom. We also optimized the chamber volume and used a syringe pump to obtain a very constant flow rate for expelling the aerosol from the chamber. In contrast to the Penn-Insufflator device, we used separate air flows for the dispersion and delivery of the aerosol to the mice, respectively. We separated these air flows because the effective dispersion of powder masses in the milligram range requires considerably higher air flows (and volumes) than those that can be endured by mice. In addition, we aimed for a higher level of precision and reproduction in the procedures, e.g., by adjusting the aerosol emission time to the desired aerosol dose. This was based on the in vitro characterization of the flow-rate-delivered dose relationship for the formulation used. Various minor improvements may have played a role too, such as sealing the dose compartment to avoid premature powder discharge and moisture uptake by the powder.

Using β-cyclodextrin as a model compound with trileucine as a dispersion enhancer, we could show in our study that the aerosol generator was suitable for deagglomerating spray-dried powder formulations to a particle size range appropriate for lung deposition. Thanks to the classifying function of the system, some larger particles and agglomerates could effectively be retained in the aerosol chamber up to an expelling flow rate of 30 L/min, and the particle size distribution of the emitted aerosol very closely approached the primary particle size distribution of the powder. In order to achieve a high aerosol concentration (to reduce the emission time and increase the dose-administration efficiency), the volume of the aerosol chamber was reduced to approximately 100 mL. Nevertheless, it is likely that, depending on the formulation, there is an optimum between chamber volume (and thus aerosol concentration) and emitted dose. This could be elucidated in future studies. In order to obtain a reproducible emitted dose, variables such as the time interval between aerosol generation and emission, the expelling flow rate and the total administration time were kept constant or changed in a controlled way. This enabled us to establish a relationship between the expelling flow rate and the emitted dose specific to the formulation used in this study. With this relationship, we could accurately compute the necessary settings (regarding the flow rate and administration time) for the emission of a desired amount of powder. By the incorporation of a fluorescent marker in the powder, we showed that, with this technique, the distribution of the powder over the entire lungs of the mice was obtained. In addition, all mice that received multiple administrations recovered well after the experiment, indicating that the applied administration procedure with the improved aerosol generator was well tolerated by the animals. 

Although many events occur in aerosol chambers after powder dispersion, including particle sedimentation and particle adhesion to the inner wall, their relative influence on the emitted dose likely depends on the flow rate. Specifically, at lower flow rates, sedimentation is expected to have a dominant influence because of the presence of a substantially higher mass fraction of particles during the runtime of the experiment in the aerosol chamber than at higher flow rates. At higher flow rates, the residence time of particles is lower, and the air circulation probably keeps more particles airborne, thereby reducing gravimetric settling. Notwithstanding this, the replicate data points in the calibration curve for our formulation showed little variation, which indicated a high reproducibility for the emitted dose. While some sedimentation was inevitable, the air stream from the inlet nozzle that was placed in the center of the top plate of the aerosol chamber (to control the aerosol emission rate) likely created a mild air circulation within the chamber during experiments that kept the particles airborne and, therefore, partly prevented sedimentation. As has been shown from the particle size measurements, the narrow size distribution of the emitted aerosol indicated that only larger particles settled in the chamber quite rapidly. By the action of gravity, particles with a density of 1 g/cm^3^ and an aerodynamic diameter larger than 10 μm travelled in less than 25 s from the top to the bottom of the 7.8 cm high aerosol chamber and stayed there, even at a relatively high flow rate of 30 L/min [25]. For that reason, they were retained in the aerosol chamber, preventing them from being delivered to the mice. 

The high reproducibility of the emitted dose under the defined conditions, as shown in Figure 4 and Figure 5, provided a much better control over the aerosol emission than is possible with the Penn-Century Insufflator device. In addition, at the low flow rate of approximately 5 mL/min, only particles smaller than 5 μm were administered to the mice. This increased the chance of better (peripheral) airway penetration. Yet, and in spite of all these improvements, we observed a substantial variation between the mice regarding aerosol deposition and distribution (Figure 7c). This advocates for making a distinction between aerosol emission on the one hand and deposition in the lungs of the mice on the other. It also suggests that the actual distribution of the powder over the mouse lung was influenced by external factors that contributed to biological variation, including the breathing (frequency) of the animals and potential losses through backflow of the aerosol to the outer environment. 

Mice have an average total lung volume of only 740 μL [7], a tidal volume of approximately 130–200 μL and a very high breathing frequency (160–200 min^−1^), depending on the mouse strain [7,8,9,26,27]. To our knowledge, no studies have thus far described the effect of supplying the lungs with a continuous airflow of 5 mL/min (83 μL/s). As 83 μL is more than 10% of the total lung volume, this may have created a pulsating overpressure in the lungs depending on the diameter of the cannula relative to that of the trachea that resulted in a backflow (of air laden with aerosol) past the cannula to the environment. Indeed, we observed the retention of the powder in the trachea. Also, we noted the presence of a fluorescent signal at the nose tip of the animals (Supplementary Figure S2). In addition, we observed, during aerosol administration, an increase in the breathing frequency of the animals, which may be indicative of them trying to compensate for the induced overpressure. This made the delivered lung dose uncertain, in spite of a fairly well-controlled aerosol supply (rate) via the cannula. The finding that certain mice showed a lower total lung deposition than others may also relate to a larger distance between the tip of the cannula and the bronchi. Therefore, further research into the (controllable) variables that influence the site of deposition in mice upon receiving a continuous aerosol supply through a cannula is recommended. 

In a recent study, it has been proposed to supply the cannula with a flexible image fiber connected to a light source and a complementary metal–oxide–semiconductor camera in order to control by visualization the exact position of the cannula tip [28]. This system can be used for the delivery of wet and powder aerosols, and its combination with an aerosol generator that emits a well-controlled powder aerosol regarding dose and size distribution might be a significant improvement in the administration of aerosols to rodents. Besides this, it is recommended that techniques be developed and improved to accurately analyze real deposition masses in mouse lungs. At least this will enable the assessment of the effect of random deposition and distribution patterns. Nevertheless, it should be realized that the variation in the lung deposition observed between the mice was not that different from the biological variation in the lung deposition after dry powder inhalation in humans (see, for example, a study by Newman et al. [29]). So, in that respect, the current device may hold good predictive properties for the situation in humans.

## 5. Conclusions

The developed aerosol generator enables the reproducible administration of predefined powder doses to the lungs of small laboratory animals. However, the precise control of aerosol deposition remains difficult. As the actual amount of powder that deposits in the lungs is likely dependent on several factors that contribute to the (biological) variation, including the positioning of the cannula in the trachea (i.e., relative to the carina and the bronchi) and the animal’s breathing, it may significantly deviate from the emitted dose. Because such variation is partly inevitable, we believe that future research should aim to further improve the emitted dose at low flow rates by finding the optimum between the aerosol concentration and sedimentation-induced losses. In addition, the versatility and robustness of the system could be assessed for other formulations as well, such as adhesive mixtures, potentially varying the dispersion principle according to the formulation that is envisioned.

## Figures and Tables

**Table 1:** Primary particle size distribution (PSD) of the spray-dried particles and size distribution of the aerosol emitted by the aerosol generator, as measured by laser diffraction. The distributions are represented by the mean X10, X50 and X90 values ± standard deviation (the latter only for the primary particle size). In addition, the fraction of the powder < 5 µm is shown. n = 3 for the primary particle size measurements; n = 2 for the PSD of the emitted aerosol.

	X10 (µm)	X50 (µm)	X90 (µm)	% <5 µm
Primary PSD	0.74 ± 0.00	1.87 ± 0.02	4.09 ± 0.03	95.8
PSD of emitted aerosol	0.71	1.60	3.00	100

## Data Availability

Original data are available on request. Please contact the corresponding author.

## Supplementary Materials

The following supporting information can be downloaded at: https://www.mdpi.com/article/10.3390/pharmaceutics15071847/s1. Supplementary Method S1: Aerosol generator assembly and operation; Figure S1: Aerosol generator parts; Figure S2: SEM images of the powder formulation; Figure S3: Fluorescence images of the whole body of mice after powder administration; Figure S4: Fluorescence images of the extracted lungs of mice after powder administration; Figure S5: Fluorescence images of the dissected lungs of mice after powder administration; Video S1: illustrative movie of the aerosolization process by the aerosol generator; Video S2: aerosol emission from the aerosol generator.
