# Supplementary material for: Performance Testing of a Homemade Aerosol Generator for Pulmonary Administration of Dry Powder Formulations to Mice
Source: Pharmaceutics. 2023 Jun 28;15(7):1847. doi: 10.3390/pharmaceutics15071847 (PMC10385055; doi:10.3390/pharmaceutics15071847)
Supplement: Supplementary file 1 [file pharmaceutics-15-01847-s001.zip › pharmaceutics-2429712-supplementary.pdf]

Supplementary materials

1. Supplementary methods

1.1. Aerosol generator assembly and operation

Figures S1a-c show the base of the aerosol generator that holds the blister with 50 mg of spray dried powder (Figure S1a). The position is such that one end of the oblong blister is vertically in line with the centrally placed disperser after complete assembly of all parts. The blister is sealed (Figure S1b) with a long aluminum lidding foil that extends beyond the outer diameter of the aerosol generator (Figure S1e,f). Sealing of the blister is necessary as it prevents premature powder release when the negative pressure is created in the aerosol chamber. The blister is covered with a plate-like part that contains a valved air inlet channel towards the eccentrically placed blister end (Figure S1c). This valve (A) is closed during the phase of creating a vacuum in the aerosol chamber and first opened to let air in for the aerosol creation. Further assembly of the parts includes subsequently placing the disperser (Figure S1d), the base of the aerosol chamber (Figure 1e, having a discharge opening for the disperser), the cylindrical wall of the aerosol chamber, with (Figure S1f) or without concentric cylinders to decrease its volume, and finally the cover plate of the aerosol chamber (Figure S1g). This cover plate has a valved connector (B) for the vacuum pump and an air inlet valve (C) used for expelling the aerosol at a controlled rate with a syringe pump. The expelled aerosol leaves the aerosol chamber via a valved outlet port (D) that is connected to the base of the aerosol chamber (Figure S1g). For this study, we used a Welch WOB-L® 2522Z-02 piston vacuum pump (Welch vacuum, Fürstenfeldbruck, Germany) to create the desired pressure in the aerosol chamber (approximately 90 kPa below atmospheric pressure). After reaching the desired pressure, valve B (to the vacuum port) is closed. Immediately after opening valve A, the aluminum lidding strip protruding from the aerosol generator is pulled away to let the air flow into the system and to entrain the powder through the disperser to create the aerosol that is stored in the aerosol chamber. For emitting the created aerosol, a NE-300 syringe pump (ProSense B.V.) is used with a 60 mL syringe (Codan B.V.).

2. Supplementary figures

Figure S1: Aerosol generator parts

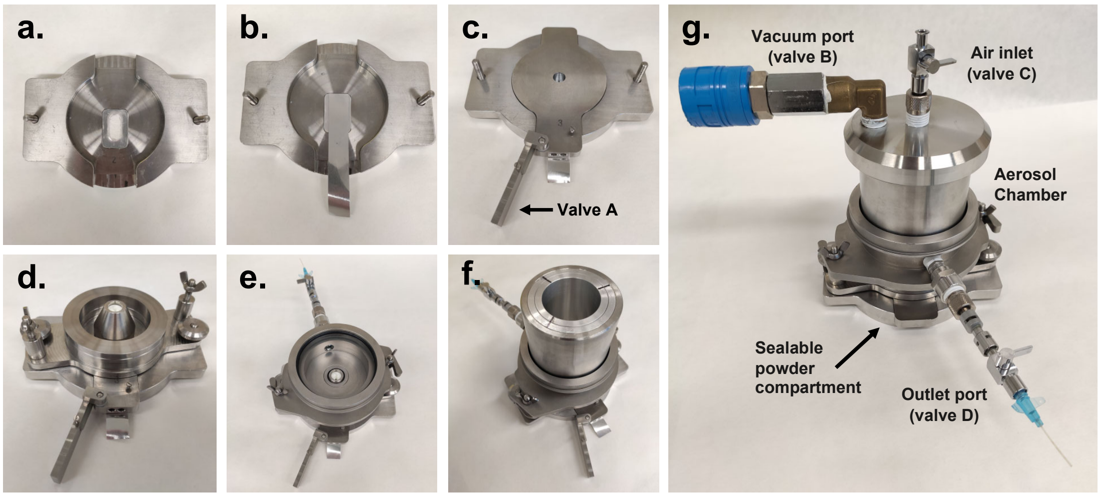

Figure S1: Aerosol generator parts. (**a**) Base plate containing the open aluminum blister cup with powder. (**b**) powder cup sealed with an aluminum lidding strip. (**c**) intermediate plate with air inlet and internal channel to the blister and valve A. (**d**) part carrying the tortuous channel of the Turbuhaler® as disperser. (**e**) base of the aerosol chamber with opening for the disperser outlet. (**f**) cylindrical walls of the aerosol chamber shown with two insert cylinders. (**g**) top plate of the aerosol chamber with the vacuum port (valve B), air inlet port (with valve C) and aerosol outlet port (with valve D).

Figure S2: scanning electron micrographs

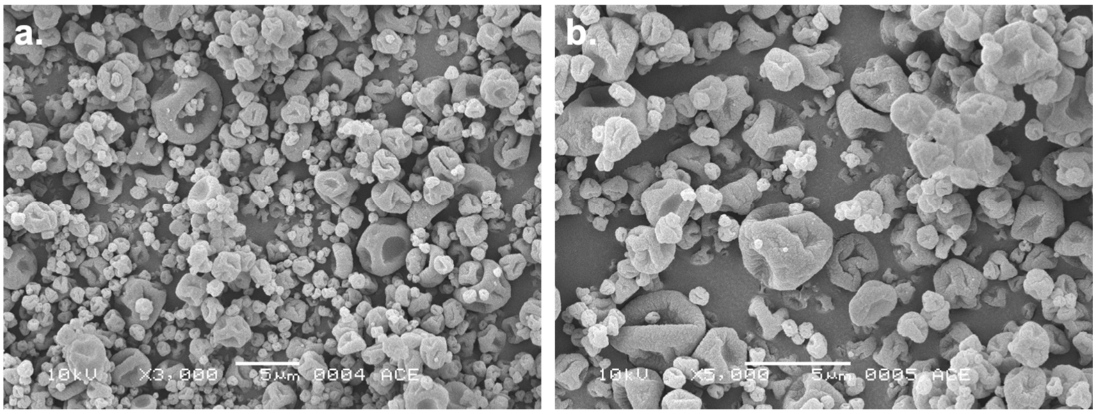

Figure S2: SEM images of the powder formulation showing wrinkled particles with a slightly rugose surface. (**a**) powder view at a 3000x magnification. (**b**) 5000x magnification.

Figure S3: Fluorescence images of the whole body of mice after powder administration

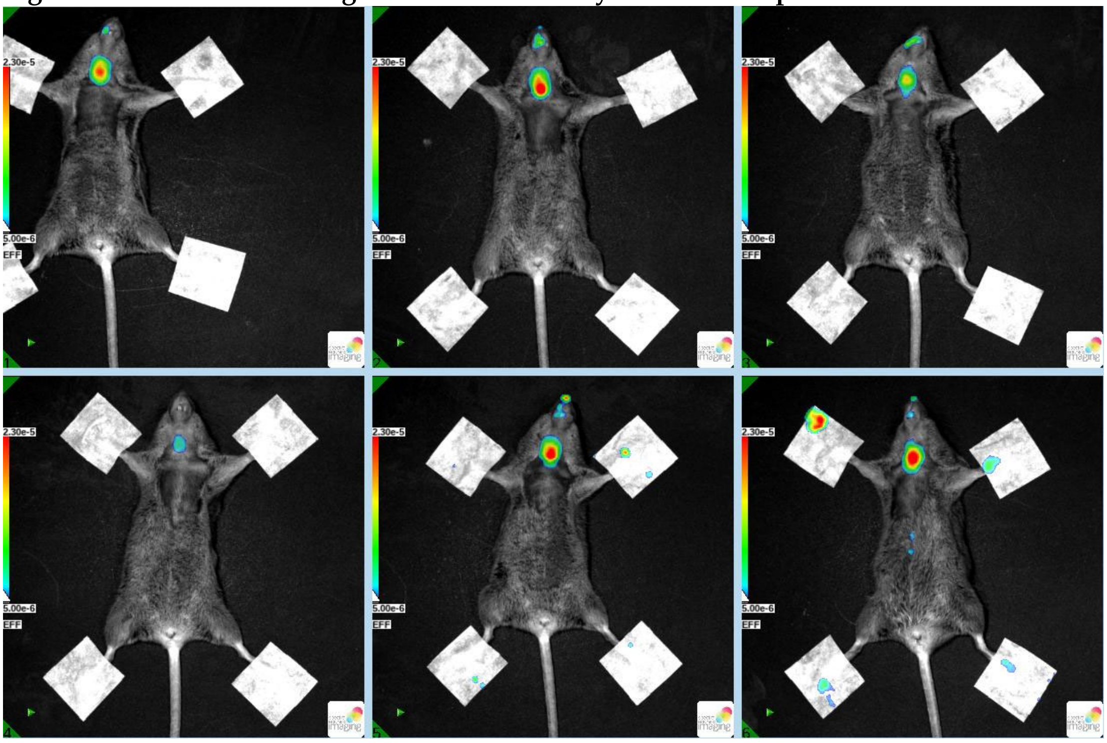

Figure S3: Ventral view of the whole body of 6 mice receiving the ICG-labeled powder formulation via the aerosol generator. Mice were treated at a fixed flow rate of 5.01 mL/min for 3 minutes and immediately imaged thereafter. The signal represents fluorescence efficiency values.

Figure S4: Fluorescence images of the extracted lungs of mice after powder administration

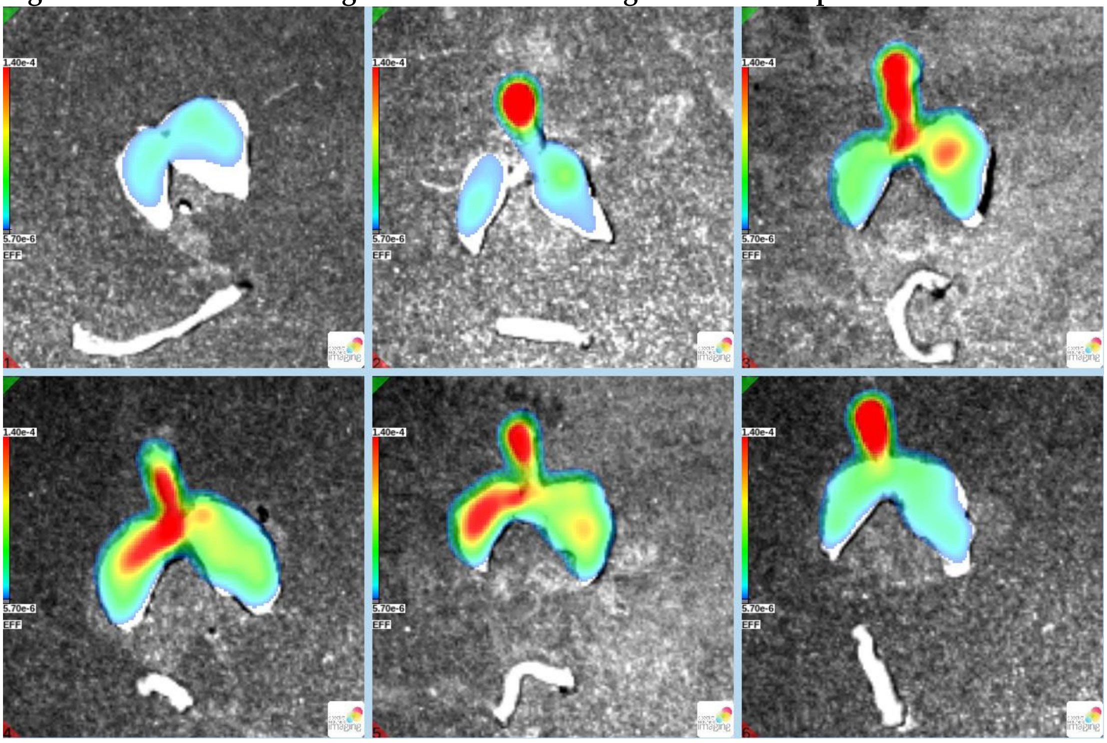

Figure S4: dorsal view of dissected lungs from each mouse with the colon serving as internal control for background fluorescence. It can be seen that lung distribution has been achieved in all animals. Data were normalized to the animal that showed the highest fluorescence intensity (mouse 4). The image corresponding to mouse 1 did not include the trachea. n=6.

Figure S5: Fluorescence images of the dissected lungs of mice after powder administration

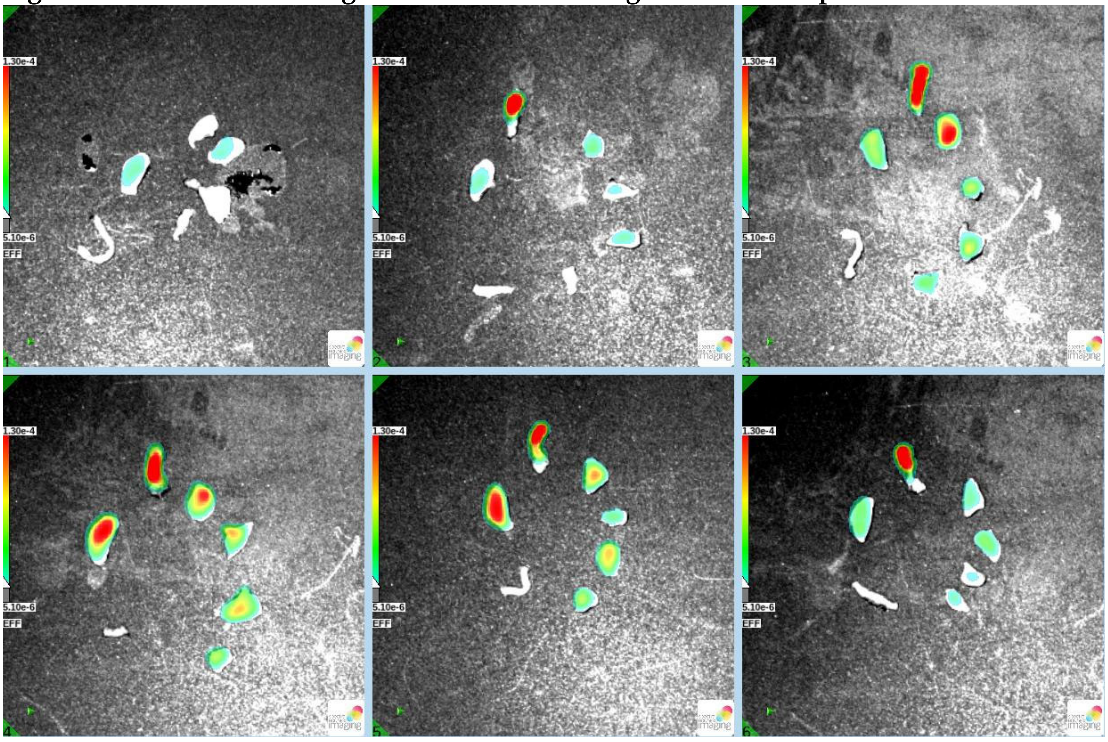

Figure S5: dorsal view of dissected lung lobes from each mouse with the colon serving as internal control for background fluorescence. Lung lobes were classified as follows, starting from the top: trachea, superior lobe, middle lobe, inferior lobe, post-caval lobe and left lobe. It can be seen that lung distribution has been achieved in all animals. Data were normalized to the animal that showed the highest fluorescence intensity (mouse 4). The image corresponding to mouse 1 did not include the trachea. n=6.
